# Glucagon-like peptide-1 receptor agonism improves lung cancer outcomes and tumor growth control

**DOI:** 10.1172/jci.insight.195484

**Published:** 2025-08-26

**Authors:** Akhil Goud Pachimatla, Bailey Fitzgerald, Joyce Ogidigo, Meera Bhatia, Randall J. Smith, Kalyan Ratnakaram, Sukumar Kalvapudi, Yeshwanth Vedire, Deschana Washington, Robert Vethanayagam RR, Hua-Hsin Hsiao, Spencer Rosario, Viraj R. Sanghvi, Joseph Barbi, Sai Yendamuri

**Affiliations:** 1 Department of Thoracic Surgery, Roswell Park Comprehensive Cancer Center, Elm and Carlton Streets, Buffalo, New York, USA.; 2 Department of Medical Oncology, Roswell Park Comprehensive Cancer Center, Buffalo, New York, USA.; 3 Department of Medicine, Herbert Irving Comprehensive Cancer Center, Columbia University Irving Medical Center, New York, New York, USA.; 4 Department of Immunology, Roswell Park Comprehensive Cancer Center, Elm and Carlton Streets, Buffalo, New York, USA.; 5 Department of Biostatistics and Bioinformatics, Roswell Park Comprehensive Cancer Center, Buffalo, New York, USA.; 6 Naomi Berrie Diabetes Research Center, Columbia University Irving Medical Center, New York, New York, USA.

**Keywords:** Clinical Research, Oncology, Drug therapy, Lung cancer, Obesity

## Abstract

BACKGROUND. Emerging evidence indicates a reduced incidence of multiple cancers in users of glucagon-like peptide-1 receptor agonists (GLP-1RAs), drugs widely used for glycemic control and weight reduction that modulate several key regulators of metabolism. We sought to examine their association with non–small cell lung cancer (NSCLC) outcomes in overweight and obese patients and gain mechanistic insights from mouse models.

METHODS. Two clinical cohorts of overweight and obese patients with NSCLC — one undergoing surgical resection (*n* = 1,177, 71 GLP-1RA users) and another receiving immune checkpoint inhibitors (*n* = 300, 10 GLP-1RA users), were propensity score matched for relevant covariates and analyzed for clinical outcomes.

RESULTS. GLP-1RA use was associated with increased recurrence-free survival in overweight and obese patients (HR: 0.41, 95% CI: 0.16–1.04, *P* = 0.026) after lobectomy. GLP-1RA treatment reduced tumor burden in obese but not normal-weight mice and altered the frequency and phenotypes of leukocyte populations and gene expression patterns in obese tumors, crucial to cancer progression and antitumor immunity. Concurrent GLP-1RA and immunotherapy was also associated with improved overall (HR: 0.41, 95% CI: 0.16–1.01, *P* = 0.027) and progression-free survival (HR: 0.31, 95% CI: 0.10–0.94, *P* = 0.019) for patients with advanced NSCLC.

CONCLUSIONS. In our cohort, GLP-1RAs enhanced lung cancer–specific clinical outcomes and augment immunotherapy efficacy. Preclinical evidence suggested this effect to be obesity restricted and mediated by immune modulation of the tumor microenvironment.

FUNDING. George Duke, Department of Defense (W81XWH-21-1-0377), NIH/NIGMS (GM147497), American Cancer Society (RSG-22-071-01-TBE), NIH (1R01 CA255515-01A1), NIH/NCI (P30CA013696 and P30CA016056).

## Abstract 2

GLP-1RAs may enhance lung cancer-specific clinical outcomes and augment immunotherapy efficacy. Preclinical evidence suggests this effect is obesity restricted and mediated by immune modulation of the tumor microenvironment.

## Introduction

Glucagon-like peptide-1 receptor agonists (GLP-1RAs), initially introduced as a class of antidiabetic drugs, have entered widespread use after their recent approval for the treatment of obesity and obesity-related conditions (1). Several retrospective studies further associate GLP-1RA use with altered cancer incidence, yet the underlying mechanisms remain unclear (2, 3).

Although the relationship between obesity and lung cancer risk is contentious, with some studies reporting a paradoxically reduced risk of lung cancer in obese populations (the so-called “obesity paradox”), evidence generated by our group and others now supports a correlation between excess visceral fat and obesity-related lung cancer risk (4, 5).

Visceral adiposity induces multiple changes in metabolic pathways, resulting in chronic meta-inflammation and suppression of antitumor immunity. Specifically, it is known to hamper the function of CD8^+^ T cells (6), key mediators of the immune response against cancer, while broadly upregulating the expression of inhibitory immune checkpoint molecules such as programmed cell death protein 1 (PD-1) (7) and enhancing suppressive leukocyte populations (8). Previous studies demonstrate that metformin, another common antidiabetic agent, can reverse multiple obesity-associated immune disturbances and improve lung cancer outcomes in overweight and obese individuals (9). Given that GLP-1RAs are effective in promoting weight loss and reducing visceral adiposity (10), we hypothesized that GLP-1RAs may similarly counteract the detrimental effects of obesity on antitumor immunity and disease progression, leading to improved non–small cell lung cancer (NSCLC) outcomes.

To test our hypothesis, we first interrogated the in vitro effects of the GLP-1RA liraglutide on murine lung cancer cell lines. We then examined the effects of distinct GLP-1RA drugs on tumor growth dynamics in vivo using both implantable and KRAS-mutated, autochthonous murine lung cancer models, scrutinizing the effects of GLP-1RA treatment on the immune tumor microenvironment (TME). Finally, in an exploratory analysis, we performed 2 retrospective analyses of real-world single-center outcome data for patients with NSCLC treated with GLP-1RAs, first in a postsurgical setting, and then in advanced disease in combination with immune checkpoint inhibitors (ICIs).

## Results

### Liraglutide does not impair murine lung cancer cell proliferation in vitro.

We investigated the in vitro effect of liraglutide on *Kras^lox-stop-lox(lsl)-G12D/+^*; *Tp53^fl/fl^*-NINJA (KPN1.1) and Lewis lung carcinoma (LLC) cell proliferation. Based on reported steady-state plasma concentrations of liraglutide ranging from 20–40 nM for typical clinical doses (0.6–1.8 mg), we examined a range of concentrations: 0, 10, 30, 90, 270, and 810 nM. Cell proliferation was assessed by measuring the fold change in absorption values after 48 hours of treatment. There was no significant difference in cell proliferation for KPN1.1 cells across liraglutide concentrations, while LLC cells exhibited increased proliferation at higher drug concentrations (Supplemental Figure 1, A and B; supplemental material available online with this article; https://doi.org/10.1172/jci.insight.195484DS1).

### Liraglutide inhibits the progression of implanted tumor growth in obese, but not normal-weight mice.

**Figure 1:**
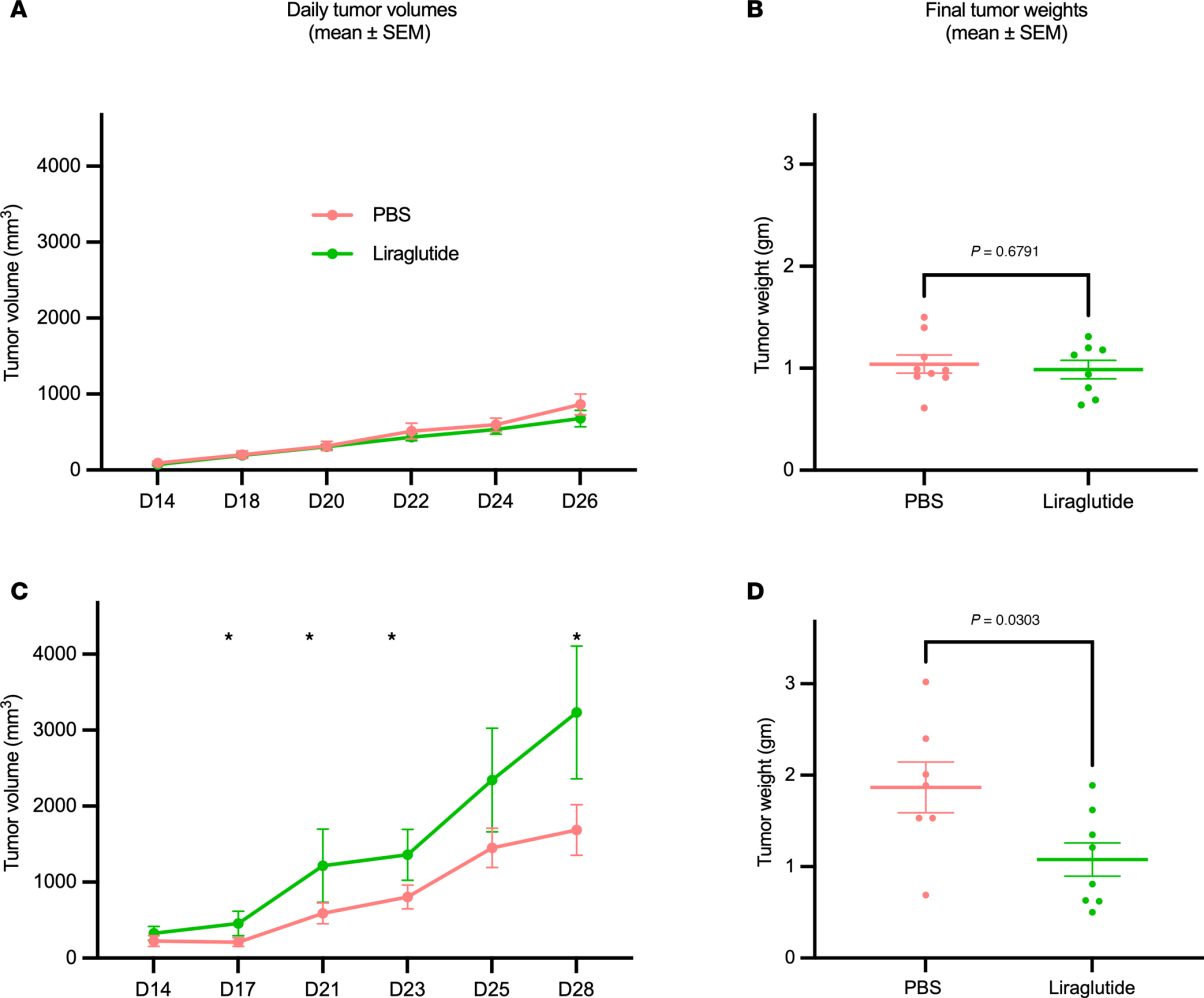
Liraglutide suppresses tumor growth in mice with diet-induced obesity. KPN1.1 cell lines (7.5 × 10^4^ cells each) were injected subcutaneously in the flanks of normal-weight (*n* = 8/group) or obese (*n* = 8/group) C57BL/6J mice fed a control or high-fat diet for 12 weeks, respectively. Once tumors were palpable, mice received daily intraperitoneal injections of liraglutide or vehicle (PBS). The volumes and weights of tumors in normal-weight mice were not significantly different (**A** and **B**). Tumors induced in obese mice had significantly higher volume and end weight in control mice than in liraglutide-treated mice (**C** and **D**). Shown are representative findings from at least 2 independent experiments (*n* = 8 mice/group/trial). *P < 0.05, results of unpaired *t* test.

We next investigated the effect of liraglutide on tumor growth in vivo. Here, normal-weight (lean) C57BL/6J mice and those with diet-induced obesity (DIO) were challenged with subcutaneous (s.c.) injection of KPN1.1 lung cancer cells. Upon detection of palpable s.c. tumors, mice received daily liraglutide (0.2 μg/g body weight) or a PBS vehicle intraperitoneally. While liraglutide-treated mice exhibited a slight decrease in body weight during the experiment, and control mice showed an increase (weight change: –0.78 g vs. +0.98 g, respectively; Supplemental Figure 2, A and B), no statistically significant difference was observed in mean tumor volumes or final tumor weight (0.98 g vs. 1.04 g; *P* = 0.67) in normal-weight/lean mice (Figure 1, A and B). In contrast, liraglutide administration significantly inhibited the progression of tumor growth, as evidenced by reductions in tumor volumes and weight (1.07 g vs. 1.86 g; *P* = 0.03) relative to the vehicle group (Figure 1, C and D). Of note, liraglutide-treated DIO mice demonstrated greater weight reduction compared with their respective controls (13.46 g vs. 4.06 g, respectively; Supplemental Figure 2, A and B).

### RNA sequencing reveals GLP-1RA–altered protumor gene expression in mice with implanted lung cancers.

**Figure 2:**
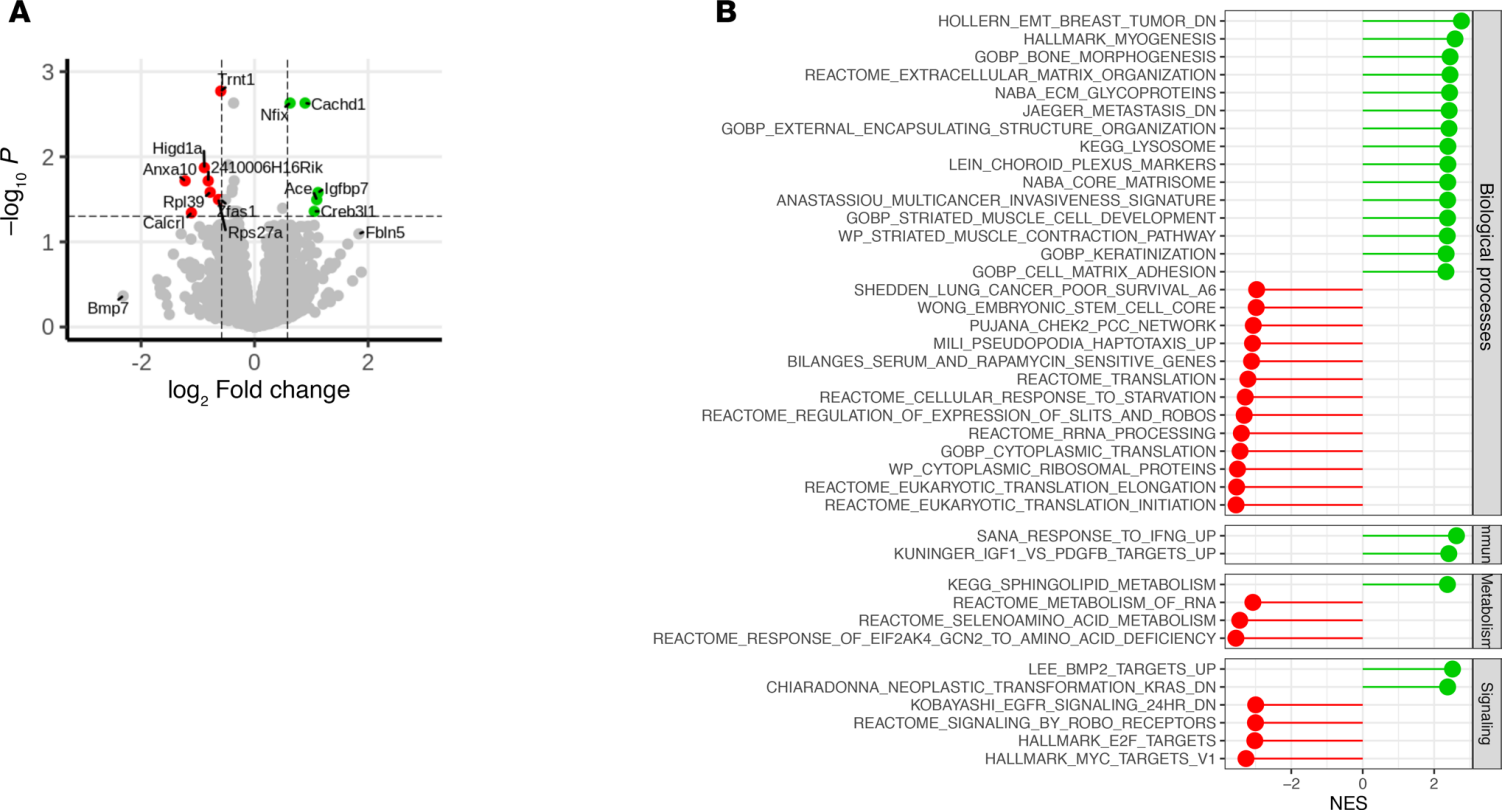
Gene expression changes in tumor cells from obese mice and altered pathways with liraglutide treatment. (**A**) Volcano plot: Differentially expressed genes in liraglutide-treated (green) versus vehicle-treated tumors (red). The *x* axis shows log_2_(fold change); the *y* axis shows statistical significance, which was assessed using ANOVA with Benjamini-Hochberg correction. (**B**) Changes in biological processes, immune, cell signaling, and metabolic pathways, as seen by changes in gene sets, expressed as normalized enrichment score (NES).

To gain insight into the mechanism underlying the benefits of GLP-1RAs suggested by clinical and preclinical studies, tumor gene expression was compared in liraglutide- and vehicle-treated s.c. tumors harvested in DIO mouse model studies using RNA sequencing (RNA-Seq). Of the 35,141 transcripts assessed, 27 were differentially expressed significantly (adjusted *P* < 0.05 and |logFC| > 0.58, Figure 2A). The transcripts upregulated with liraglutide treatment (Figure 2A, green) included *Igfbp7*, which induces G_1_ phase cell cycle arrest by upregulating cyclin-dependent kinase inhibitors. Additionally, treatment was associated with increased *Creb3l1*, a regulator of angiogenesis (11), and *Nfix*, which encodes a factor with pro- and antitumor roles that are also involved with immune cell development and differentiation (12). Conversely, liraglutide exposure was also associated with decreased *Anxa10*, a well-known marker of poor prognosis with a role in various cell signaling pathways (13). Furthermore, the drug was linked to reduced expression of the protumor genes *Higd1a* and *Rpl39*, associated with hypoxia-induced cellular stress and metabolism, and cell proliferation and migration, respectively (14, 15).

Gene set enrichment analysis (GSEA) revealed significant treatment effects on several pathways relevant to cancer outcomes (normalized enrichment score > 2, *q* < 0.05) spanning cell proliferation, cellular respiration, immune signaling, and cell signaling (Figure 2B). Notably, genes linked to signaling triggered by IFN-γ (an antitumor immunity–promoting cytokine) and growth factor (insulin-derived growth factor) signaling were significantly upregulated with liraglutide treatment. Several metabolic pathways important for tumor and leukocyte biology, including fatty acid (sphingolipids) and amino acid metabolism, were enhanced and disrupted, respectively, and genes important for translation-associated processes were also apparently suppressed with treatment, as were those associated with poor lung cancer survival. Finally, multiple cell signaling pathways well appreciated for driving tumor development and progression were also suppressed in GLP-1RA–treated tumors, including those involving *KRAS*, *EGFR*, *MYC*, and *E2F* transcription factors (Figure 2B). Overall, these findings link liraglutide with gene expression changes in the tumor relevant to immunity, metabolism, and tumor progression that are likely to reflect or explain the effects of GLP-1RAs seen in mice and patients.

### GLP-1RA treatment enhances antitumor immune responses in mice.

GLP-1R is expressed in several leukocyte populations (16), but the effects of GLP-1RAs on these cells are apparently complex, and their likely impact on the antitumor immune response is unclear. While many have found the effects of GPL-1R signaling and agonism to be immunosuppressive (17), some studies suggested enhanced antitumor immunity (18, 19). To explore whether liraglutide’s antitumor effects in our mouse model studies stem from favorable modulation of antitumor immunity, we used flow cytometry to characterize the phenotypic and functional changes among the tumor-infiltrating leukocytes (TILs) associated with treatment in harvested s.c. tumors.

**Figure 3:**
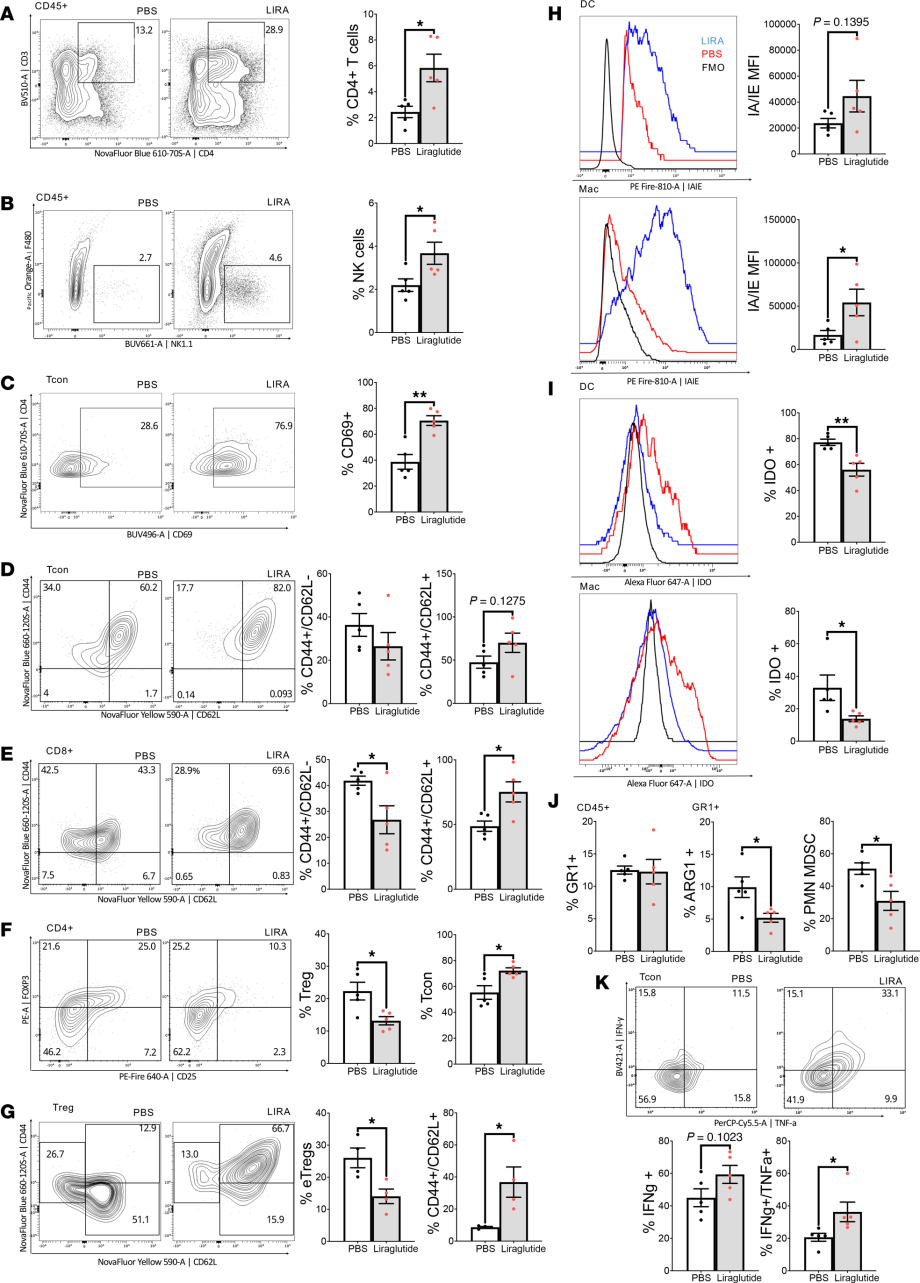
Characterizing the impact of GLP-1RA treatment on tumor-infiltrating leukocyte populations and phenotypes. Tumor tissues were recovered from the obese mice described in Figure 2, and, after enzymatic digestion, single-cell suspensions were generated. Immunostaining followed by spectral flow cytometry analysis revealed the effects of liraglutide (LIRA) on T cell (**A**–**G**) and myeloid cell (**H**–**J**) populations. Flow analysis was also performed to assess intracellular cytokine production by leukocytes restimulated ex vivo with PMA/ionomycin and brefeldin A (**K**). Shown are the mean ± SEM results from 1 of 2 independent experiments and representative flow plots (*n* = 5 per group/trial). *P < 0.05, **P < 0.02 by *t* test.

Increased CD4^+^ T cell and natural killer (NK) cell frequencies in the tumors of GLP-1RA–treated obese mice were seen (Figure 3, A and B). Proportions of conventional (i.e., non-regulatory) CD4^+^ T cells (Tcon), marked by the activation marker CD69, were also elevated in liraglutide-treated mice (Figure 3C), potentially indicating an enhanced antitumor immune response. Interestingly, the fraction of intratumor CD8^+^ and Tcon compartments with an effector surface marker profile (CD44^hi^CD62L^lo^) was apparently reduced in favor of CD44^+^CD62L^+^ T cells (Figure 3, D and E), suggesting a potential shift toward a memory-like phenotype in these cells.

Since Foxp3^+^ regulatory T cells (Tregs) express relatively high levels of GLP-1R (20), and a role for its downstream signaling has been reported to maintain these suppressor cells in the periphery (21), we assessed liraglutide-triggered changes among tumor Tregs. Surprisingly, proportions of Foxp3^+^CD25^+^ cells within the TIL CD4^+^ pool were significantly reduced with treatment (Figure 3F). Interestingly, the functionally potent, activated (CD44^+^CD62L^–^) or “eTreg” subpopulation made up a smaller portion of the TIL-Treg pool from liraglutide-treated mice compared with vehicle-treated control mice, in favor of a population with memory-like potential (i.e., CD44^+^CD62L^+^ surface staining) (Figure 3G). In agreement with a prior study (20), GLP-1RA treatment upregulated PD-1 and PD-L1 on tumor Tregs (Supplemental Figure 3, A and B); however, increased levels of mTOR activity (indicated by phosphorylated mTOR^+^ [p-mTOR]^+^ Foxp3^+^ T cells) and Tbet expression were found among GLP-1RA–treated Tregs as well (Supplemental Figure 3, C and D). This combination indicates an activated but unstable Treg phenotype (22–25), less capable of restricting robust antitumor immune response suppression. Collectively, these results suggest the ability of GLP-1RAs to undermine immunosuppression in obese mice.

Additional effects of GLP-1RA treatment were evident in antigen-presenting cell (APC) populations in the tumor niche. Dendritic cells (DCs) and macrophages from treated mice displayed upregulated MHC II levels, an effect that approached and achieved statistical significance in these populations, respectively (Figure 3H). Conversely, TILs of the GLP-1RA–treated mice harbored markedly fewer APCs producing indoleamine 2,3-dioxygenase (IDO) (Figure 3I), a mediator of tumor-associated immunosuppression and a mechanism of Treg expansion and differentiation (26, 27). The mice treated with liraglutide also displayed a relative scarcity of arginase-expressing myeloid-derived suppressor cells (MDSCs) (Figure 3J). Thus, it is possible that the GLP-1RA triggers APC phenotypes poised to effectively stimulate T cell responses and cell-mediated antitumor immunity. Further supporting this notion, the pool of TIL-isolated CD4^+^ T cells recovered from liraglutide-treated mice capable of producing proinflammatory, tumoricidal cytokines ex vivo was expanded (Figure 3K). In all, these findings support a potent immunostimulatory effect of the GLP-1RA liraglutide impacting several leukocyte populations that may be responsible for the improved tumor growth control and clinical outcomes linked to the drug.

### Semaglutide inhibits tumor progression and improves survival in a KRAS-driven mouse lung cancer model.

**Figure 4:**
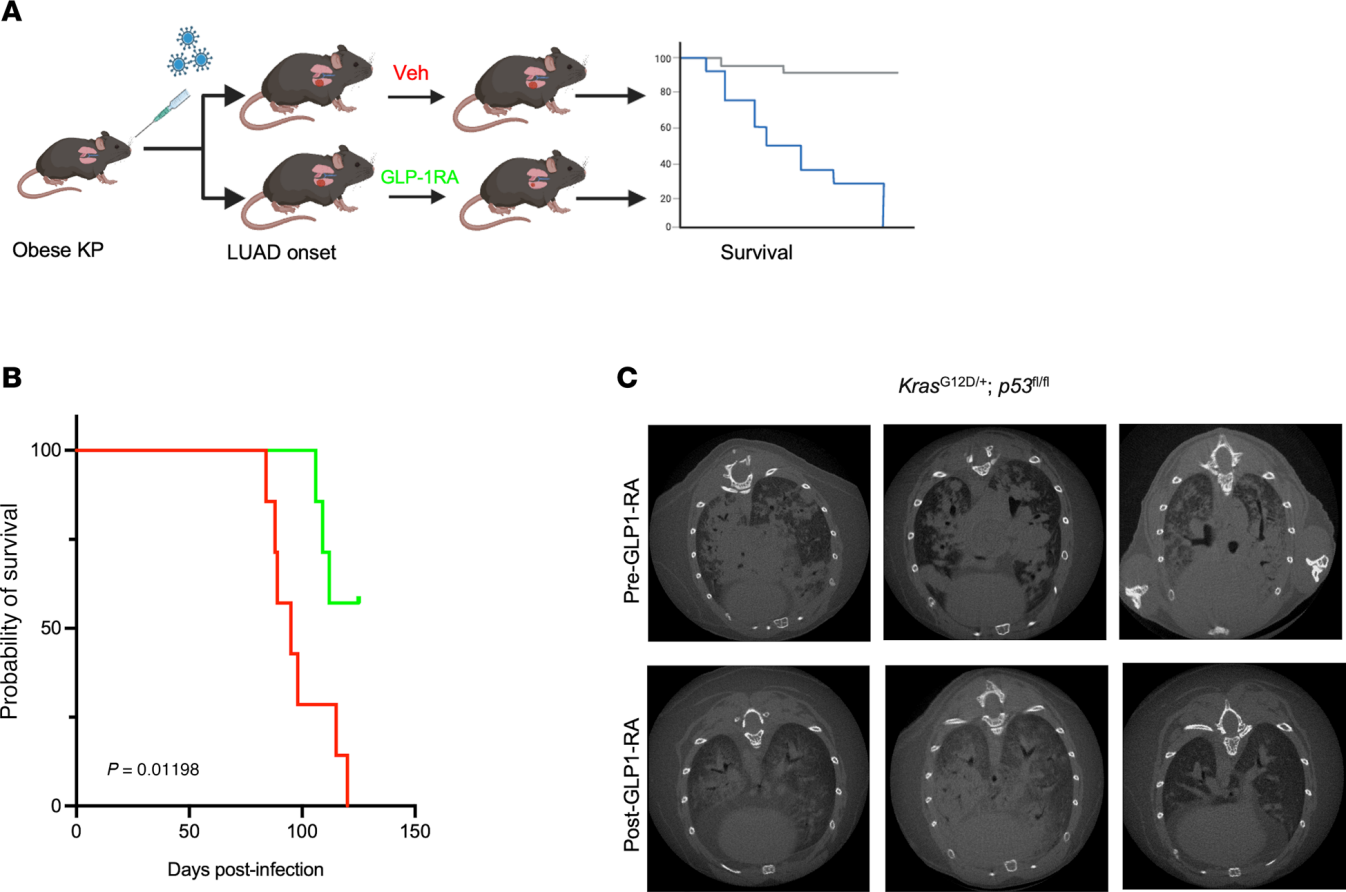
Semaglutide effect in an autochthonous mouse model. (**A**) Schematic illustrating the experimental design in the diet-induced obesity (DIO) KP mouse. (**B**) Kaplan-Meier survival analysis of DIO-KP mice receiving vehicle or semaglutide following tumor initiation (*n* = 7 per group). *P* = 0.01198 by log-rank test (vehicle vs. semaglutide). (**C**) Representative μCT images of lung tumor burden in DIO-KP mice at endpoint, comparing semaglutide- and vehicle-treated groups.

To reduce the impact of model-specific or drug-specific factors on tumor dynamics, we repeated our experiment examining the effects of GLP-1RA administration on murine tumor growth, this time in an independent autochthonous mouse model with conditional *KrasG12D* activation and *Trp53* deletion (KP) using the GLP-1RA semaglutide. To induce obesity, tumor-free KP mice were prefed a high-fat Western diet (60% kcal from fat) supplemented with sugar water (23.1 g/L D-fructose and 18.9 g/L D-glucose) for 13 weeks prior to tumor initiation (Figure 4A). Lung adenocarcinoma was initiated via intratracheal delivery of Cre recombinase; this precipitates spontaneous tumor growth, which more closely mimics the geographic pattern of human lung cancer metastases compared with s.c. implantation. The KP mice with DIO and induced lung adenocarcinoma were treated with semaglutide (*n* = 7) or vehicle control (*n* = 7).

Semaglutide treatment significantly extended overall survival (HR: 0.21, 95% CI: 0.057–0.79, *P* = 0.012) compared with vehicle-treated control (Figure 4B). Longitudinal microcomputed tomography (μCT) revealed significantly reduced tumor burden in the semaglutide groups (Figure 4C), mirroring the results of our heterotopic models, where GLP-1RA reduced tumor growth and progression in obesity-associated lung cancer.

### GLP-1RA use is associated with improved recurrence-free survival after NSCLC resection.

We then performed a retrospective examination of clinical outcomes in a database of patients undergoing surgery for NSCLC at our center between 2015 and 2024. A total of 1,177 patients met criteria for inclusion in the postsurgical cohort (a flowchart of the cohort is represented in Supplemental Figure 4A). Within this cohort, 71 patients were GLP-1RA users. Demographic characteristics, including age, clinical stage, race, and smoking history were generally well balanced between the GLP-1RA users and non-users; however, GLP-1RA users had significantly higher mean body mass index (BMI) at the time of resection than the non-users (35.07 vs. 30.6; *P* = 0.001). Demographic data are reported in Supplemental Table 1. Most patients across both groups had clinical stage I disease 970 (82.4%), while 193 (16.4%) had stage II, and 14 (1.2%) had stage III lung cancers.

In an unmatched, univariate Cox regression model including the entire cohort, GLP-1RA use was associated with improved recurrence-free survival (RFS) (HR: 0.41; 95% CI: 0.17–1.02; *P* = 0.027). No such association was noted for overall survival (OS) (HR: 0.70; 95% CI: 0.43–1.16; *P* = 0.09).

**Figure 5:**
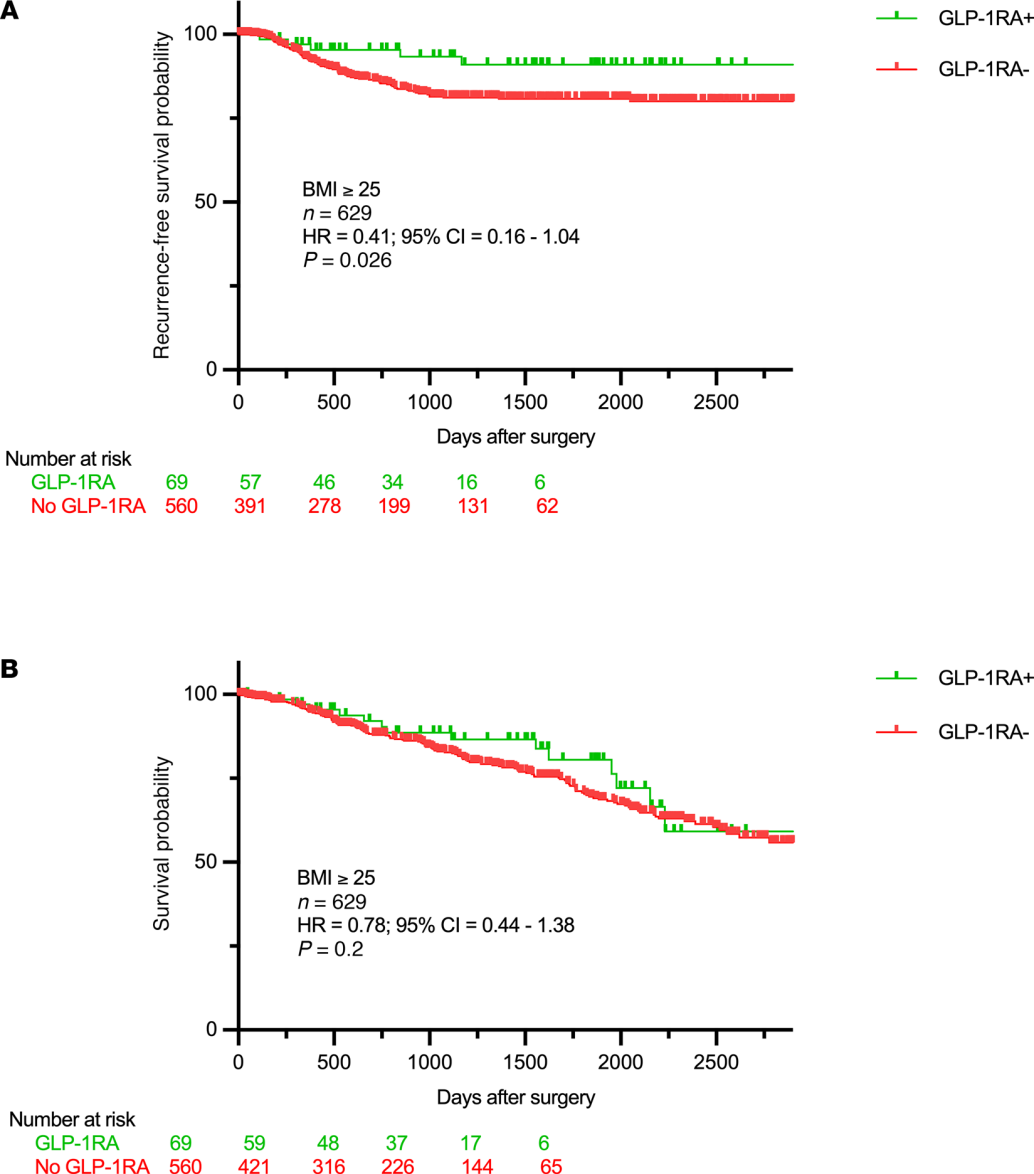
Clinical outcomes with adjuvant GLP-1RA use after resection in NSCLC. Recurrence-free survival (**A**) and overall survival (**B**) in overweight and obese patients who underwent resection for NSCLC and received GLP-1RA. GLP-1RA use is associated with improved RFS after propensity score matching for age, sex, race, smoking, histology, stage, and GLP-1RA use as covariates. Significance was assessed using Cox’s proportional hazards modeling for univariate models.

In a propensity-matched analysis, where propensity scores were calculated using age, sex, race, BMI, cancer stage, smoking status, and histology as covariates and GLP-1RA use as the dependent variable, a matched cohort of 629 was identified that included 560 GLP-1RA non-users and 69 GLP-1RA users. This cohort did not include any patients with stage III disease (see Supplemental Table 1). Univariate Cox regression of the matched cohort showed that GLP-1RA use was associated with improved RFS (HR: 0.41, 95% CI: 0.16–1.04, *P* = 0.026; Figure 5A). GLP-1RA use was not significantly associated with OS (HR: 0.78, 95% CI: 0.44–1.38, *P* = 0.20; Figure 5B). Results of univariate analyses for all the covariates in both unmatched and matched cohorts are presented in Table 1.

### GLP-1RA use improved survival in overweight patients receiving ICIs for advanced NSCLC.

In light of the findings from the preclinical model and expanding use of immunotherapy in the treatment of lung cancer, we set out to test whether the apparent benefits of GLP-1RA use extend to patients on ICI therapy. A cohort of 300 patients with advanced lung cancer treated with ICI at our center between 2015 and 2023 was identified, of which 10 patients were prescribed concurrent GLP-1RA. A flowchart of the cohort is represented in Supplemental Figure 4B. All GLP-1RA users in the cohort had diabetes (reflecting FDA-approved indications during our study period), whereas 25% of non-users were diabetic. Most patients (78.7%) had stage IV disease; the remainder of the cohort was comprised of stage III NSCLC not amenable to local treatments. Treatment included immunotherapy alone for 158 patients (52.7%) and a combination of immuno- and chemotherapy for 142 (47.3%). Patients receiving GLP-1RA were significantly younger; other characteristics such as sex, BMI, race, smoking status, tumor histology, and treatment modality were similar between groups (Supplemental Table 2).

In an unmatched analysis, univariate Cox regression modeling demonstrated that GLP-1RA use was associated with improved progression-free survival (PFS) (HR: 0.39; 95% CI: 0.13–1.14; *P* = 0.044) and OS (HR: 0.40; 95% CI: 0.15–1.04; *P* = 0.031).

**Figure 6:**
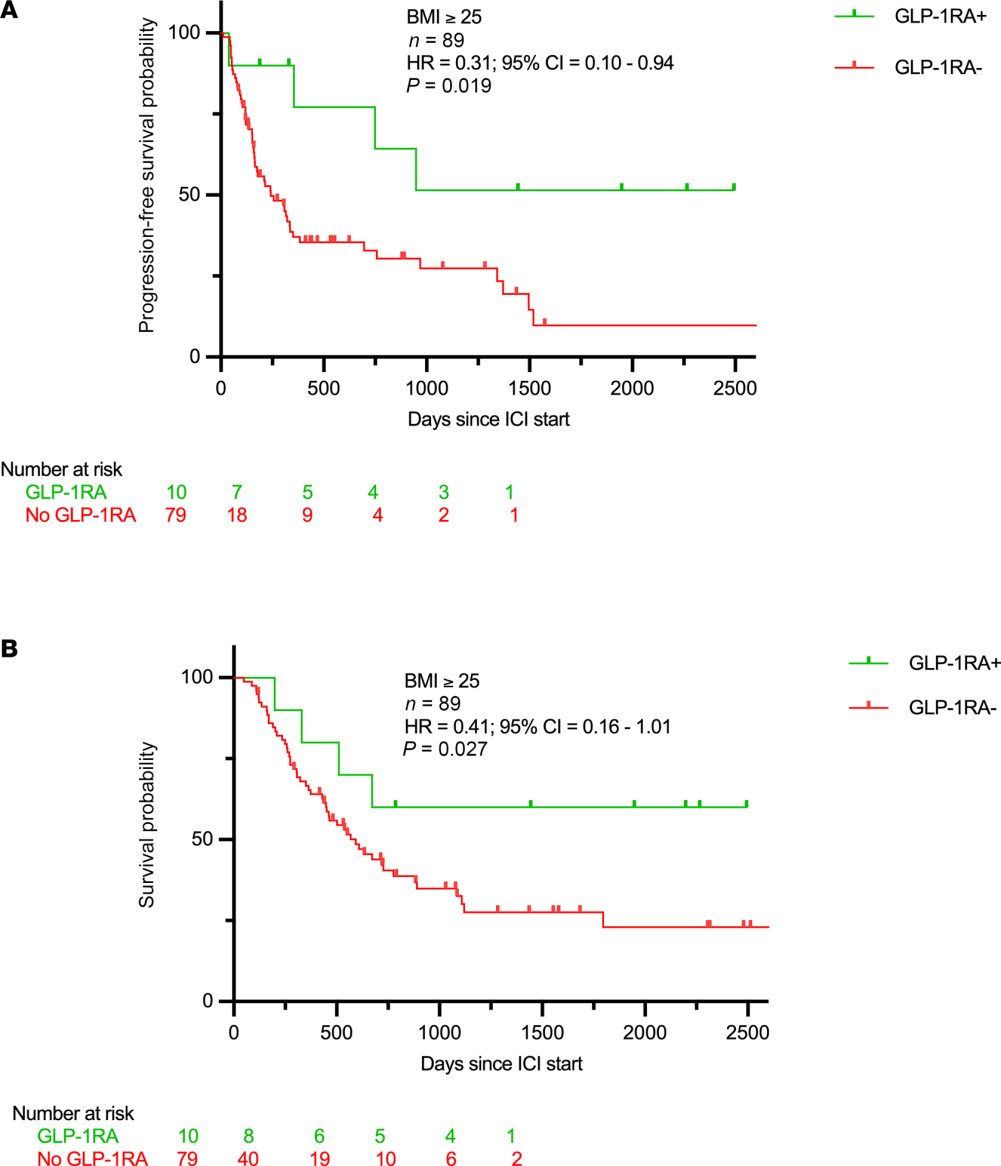
Outcomes with GLP-1RA use in advanced-stage NSCLC patients on ICI. Progression-free survival (**A**) and overall survival (**B**) in overweight and obese patients receiving ICI for advanced NSCLC after propensity score matching for age, sex, race, smoking, histology, stage, first-line therapy, and GLP-1RA use as covariates. Significance was assessed using Cox’s proportional hazards modeling for univariate models.

In a propensity-matched analysis, propensity scores were calculated using age, sex, race, smoking status, BMI, tumor histology, first-line therapy, and stage at the start of ICI therapy as covariates, with GLP-1RA use as the dependent variable. The matched cohort (*n* = 89) had 79 non-users and 10 GLP-1RA users (see Supplemental Table 2). Univariate Cox regression of the matched cohort showed that GLP-1RA use was associated with improved PFS (HR: 0.31, 95% CI: 0.1–0.94, *P* = 0.019; Figure 6A) and OS (HR: 0.41, 95% CI: 0.16–1.01, *P* = 0.027; Figure 6B). Results of univariate analyses for all covariates in both unmatched and matched cohorts are presented in Table 2.

## Discussion

The use of GLP-1RAs in the United States is rising dramatically, with expanding indications for both diabetes and obesity-related conditions (28, 29). Since these conditions also affect the majority of patients diagnosed with lung cancers (30), widespread use of these medications during treatment for lung malignancies is inevitable. Little is known about the safety of GLP-1RA use during lung cancer treatment, however, and any impact these medications may have on treatment outcomes is similarly opaque. While the effects of GLP-1RAs on the incidence of hepatic, pancreatic, colorectal, genitourinary, cutaneous, and hematologic malignancies have been reported (29), the present study marks the first attempt to our knowledge to examine the drug family’s impact on lung cancer outcomes and the potential utility of GLP-1RAs with ICI.

Unsurprisingly given our hypothesized mechanism of action, we observed little effect of GLP-1RAs on in vitro cell proliferation. These findings are in line with previous studies in the preclinical arena involving multiple cancer types that have reported inconsistent direct effects of GLP-1RAs on cancer cell proliferation (31–35), and they suggest that substantial inhibitory effects on malignant cells are unlikely. Notably, however, we observed consistent and significantly reduced tumor burden in both implanted and autochthonous mouse lung cancer models treated with GLP-1RAs. Thus, we suspect that the reduction in tumor growth seen in obese mice treated with GLP-1RAs may be linked to biological pathways active within the TME.

Indeed, our preclinical mouse studies provide some mechanistic insights in support of this notion. Parallel transcriptomic analysis substantiated an immune-stimulating effect of GLP-1RAs, revealing marked treatment-associated changes in pathways related to oncogenesis, tumor cell proliferation, and metabolism, which may modulate complex biological processes intrinsic as well as extrinsic to malignant cells themselves. Our s.c. tumor model showed that liraglutide, a widely used GLP-1RA, inhibited tumor growth selectively in obese mice and also revealed multifaceted immune-related changes in the TME consistent with enhanced antitumor immunity. These included increased CD4^+^ T cell and NK cell frequencies, bolstered pools of memory-like T cells, reduced Treg populations, and apparent improvements in APC functionality. Interestingly, changes in gene expression profiles closely resembled those observed in individuals with elevated total fat areas (36). That study, which examined genomic changes in NSCLC patients with high versus low body fat, revealed similar changes in transcripts associated with cell proliferation, signaling, and apoptosis, apart from genes regulating fatty acid metabolism. These findings indicate that liraglutide may exert its effects by modulating cellular pathways in a manner that counteracts obesity-induced metabolic adaptations, potentially leading to improved clinical outcomes.

The immunological effects we observed are notable in that they run surprisingly counter to much of the existing literature speaking to the immunomodulatory properties of GLP-1 and GLP-1RA, which generally support a role in immunosuppression in various settings. For example, GLP-1 has been convincingly characterized as a mediator of negative costimulation in T cells, with agonism leading to suppression of proliferation and allograft rejection in mouse studies. In contrast, GLP-1R antagonism, via Exendin-9-39, bolstered antitumor immunity when tested in a colorectal cancer model (37). Additional studies have characterized the activity of GLP-1RA drugs as antiinflammatory (38), and reports have suggested both high expression of GLP-1R by Treg cells (20) and a role for GLP-1 signaling in the biology of these suppressive cells (21, 39). On the other hand, our findings do resonate with a recent preclinical study that found the GLP-1RA semaglutide slows tumor growth and spread, enhances T cell responses and DC maturation, and undermines Tregs while showing no direct effects on breast cancer cell lines in vitro (19). Other studies have also suggested antitumor promise for liraglutide in a model of hepatocellular carcinoma, where it can activate NK cell–mediated responses (40). Others have found the drug can improve the antitumor effects of ICI by downmodulating additional regulators of inflammation in mouse tumor models (41, 42).

Moreover, our retrospective clinical studies provide early evidence that this effect may translate into human NSCLC. We found that in early-stage NSCLC patients undergoing surgery at our institution, GLP-1RA in the postoperative period improved RFS. Following the animal experiments and in vitro assays, analysis of GLP-1RA use in advanced-stage NSCLC patients receiving ICI demonstrated augmented benefit, with improvement in both PFS and OS. While these results are preliminary, they are suggestive and support further investigation into this drug family as a means to improve lung cancer treatment outcomes in obese patients with NSCLC.

There are several important limitations in our study that provide opportunities for future investigations. Our preclinical studies were conducted with semaglutide and liraglutide, as these drugs were the most common in clinical use prior to 2024. These drugs are both single GLP-1RA medications without co-agonism of gastric inhibitory polypeptide (GIP) and follow-up studies are needed to confirm that our results are conserved across newer dual agonist drugs, such as tirzepatide. Additionally, while our analysis of the transcriptomic and immunological impact of GLP-1RA treatment in obese tumors reveal previously uncharted effects, a definitive mechanism remains to be elucidated. It is also unclear whether the immunological changes and antitumor effects we observe stem from a direct action of the drug on leukocytes (a distinct possibility since many immune cells have been shown to express GLP-1R) or indirect effects arising from a correction of excess adiposity — a state known to oppose effective antitumor responses. However, given the incremental (albeit statistically significant) correction in obesity seen and the fact that obese mice (43) and cancer patients (44) often respond better to ICI therapy than normal-weight counterparts in the absence of weight loss, the latter possibility seems less likely to explain the benefits of GLP-1RA in our study.

We are also cautious to avoid over-interpretation of our clinical analysis, which is inherently limited by its retrospective nature and small, single-center sample size. To address this weakness and mitigate the effects of confounders in the design, we supplemented our unmatched univariate analysis with propensity score–matched analysis; however, the small numbers make multivariate analysis impossible without substantial risk of introducing type I error, so the contribution of effects such as degree of weight loss and other baseline characteristics cannot be delineated. In every case the need for prescription GLP-1RA was determined by non–oncology-treating physicians, which may introduce selection bias. Moreover, all patients within this cohort were prescribed GLP-1RA for diabetes, as this was the only FDA-approved indication during the study period. Nevertheless, as the indications for GLP-1RA treatment expand and databases including larger numbers of patients become available, further work investigating outcomes in larger populations will be worthwhile. Collectively, these limitations and lingering questions highlight the need to better understand the action of GLP-1RA modulators of metabolism and NSCLC. Such knowledge gaps provide rationale for pilot clinical trials where safety can be confirmed, and more correlative translational work can be done to compare the effects seen in our preclinical models to those in the human NSCLC TME.

While these and additional mechanistic studies are needed to fully realize the implications of our findings for future NSCLC therapy, this study provides encouraging, albeit preliminary, evidence regarding the favorable effects of GLP-1RA use after resection and during ICI treatment for NSCLC. In the context of rising obesity rates and high global lung cancer mortality, these observations may be important for adapting the treatment of lung cancer to a changing patient pool, and they warrant further investigation in the direction of mitigating obesity-associated lung cancer risk.

## Methods

### Sex as a biological variable.

Male mice were used in these lung cancer preclinical models to facilitate creation of DIO models, as male C57BL/6J mice more uniformly display significant weight gain on high-fat diets (relative to normal-diet controls) than females (45–47). This approach is consistent with established protocols in the field and facilitates direct comparison with previous studies.

### In vitro effects of liraglutide on murine lung cancer cell lines.

The murine lung cancer cell lines KPN1.1 (48) (provided by N. Joshi, Yale School of Medicine, New Haven, Connecticut, USA) and LLC (ATCC, CRL-1642-LUC2) were cultured in vitro and harvested in the logarithmic phase by trypsinization, followed by centrifugation. Cell concentration was adjusted and added to strip-well plates at 3000 cells per well, which were then incubated at 37°C. After 24 hours, the cells were treated with liraglutide at 10, 30, 90, 270, and 810 nM concentrations. To measure the cell proliferation rate, the wells were washed, and 95 μL of cell culture medium and 5 μL of CCK-8 reagent (Dojindo Laboratories) were added to each well. The strips were allowed to incubate at 37°C for 2 hours, and the absorbance value (OD value) was measured and recorded using a microplate reader at a wavelength of 450 nm at 0, 24, 48, 72, and 96 hours. The cell proliferation rate was calculated as follows: cell proliferation rate = (OD value at a given concentration at 48-hour time point – blank)/(OD value on day 0 – blank).

### Effects of GLP-1RA on murine survival, s.c. tumor dynamics, gene expression, and TME in an implanted lung cancer model.

Age-matched obese and non-obese male C57BL/6J mice were purchased (The Jackson Laboratory, strain numbers 380050 and 380056) or generated in-house by feeding normal-weight mice with high-fat (60% calories from fat, 5.4 kCal/g) or control chow (~7.2% calories from fat, 3.9 kcal/g) (Bio-Serv, products S3282 and F4031, respectively). For s.c. tumor challenge experiments, KPN1.1 cells or LLC cells were injected into the flanks of 7- to 17-week-old obese and non-obese mice (75,000 cells per mouse). Mice with palpable tumors (typically detected 6–7 days after implantation) received intraperitoneal injections of liraglutide (0.2 μg per gram of mouse body weight) or phosphate-buffered saline (PBS) vehicle daily. The mice were euthanized after 19–22 days of cell inoculation, and tissues were harvested for further analysis.

RNA sequencing was performed on excised, subcutaneous KPN1.1 tumors from obese mice treated with GLP-1RA. At the time of mouse tumor harvest, a fragment was flash frozen and was later used for RNA isolation using the miRNeasy mini kit (Qiagen). The sequencing libraries were prepared with the RNA HyperPrep Kit with RiboErase (HMR) kit (Roche Sequencing Solutions), from 500 ng total RNA. Following the manufacturer’s instructions, the first step depletes rRNA from total RNA. After ribosomal depletion, the remaining RNA was DNase digested to remove any gDNA contamination. Samples were then purified, fragmented, and primed for cDNA synthesis. Fragmented RNA was then reverse transcribed into first-strand cDNA using random primers. The next step removes the RNA template and synthesizes a replacement strand, incorporating dUTP in place of dTTP to generate double-stranded cDNA. Pure Beads (KAPA BIOSYSTEMS) were used to separate the double-stranded cDNA from the second-strand reaction mix, resulting in blunt-ended cDNA. A single “A” nucleotide was then added to the 3′ ends of the blunt fragments. Multiple indexing adapters, containing a single “T” nucleotide on the 3′ end of the adapter, were ligated to the ends of the double-stranded cDNA, preparing them for hybridization onto a flow cell. Adapter-ligated libraries were amplified by PCR, purified using Pure Beads, and validated for appropriate size on a 4200 TapeStation D1000 Screentape (Agilent Technologies, Inc.). The DNA libraries were quantified using a KAPA Biosystems qPCR kit, and were pooled together in an equimolar fashion, following experimental design criteria. Each pool was denatured and diluted to 350 pM with 1% PhiX control library added. The resulting pool was then loaded into the appropriate NovaSeq Reagent cartridge for 100-bp paired-end sequencing and sequenced on a NovaSeq 6000 following the manufacturer’s recommended protocol (Illumina Inc.). Raw reads that passed the Illumina RTA quality filter were demultiplexed and preprocessed using FastQC for sequencing base quality control. Reads were then mapped to the latest version of a mouse reference (GRCm39/mm39) using Bowtie (v1.0.1) (49) and TopHat (v2.0.13) aligner (50). Mapped reads were quantified at the gene level as a raw counts matrix using featureCounts from Subread (51). Samples were filtered after quality assessment, and a total of 10 samples were used in subsequent analyses (*n* = 5/group). Raw feature counts were normalized, and differential expression analysis was carried out using DESeq2 (52), and visualized using volcano plots. Differential expression rank order was used for subsequent GSEA (53), performed using the cluster profile package in R, and visualized via lollipop plots. Gene sets queried included the Hallmark, Canonical pathways, and GO Biological Processes Ontology collections available through the Molecular Signatures Database (MSigDB) (54).

Flow cytometry analysis was performed on the s.c. KPN1.1 tumors excised from obese mice treated with GLP-1RA. Tumors were cleaned of skin and fat before mechanical (GentleMACS dissociation) and enzymatic digestion in a collagenase/hyaluronidase mix (Stem Cell Technologies) following the manufacturer’s protocol. The resulting cell suspensions were filtered (100 μm cell strainer), washed, and pelleted before RBC lysis in 1 mL ACK buffer (Thermo Fisher Scientific). Cells were then washed with PBS containing 1% fetal bovine serum (FBS) and 2 mM EDTA before incubation with fluorochrome-conjugated antibodies diluted in like buffer. After surface immunostaining, intracellular markers (i.e., FOXP3) were stained after fixation and permeabilized using eBioscience’s FOXP3 staining kit. For intracellular cytokine staining, cell suspensions were restimulated in media containing PMA and ionomycin in the presence of GolgiPlug (BD) for 5 hours at 37°C before surface staining, fixation/permeabilization, and internal staining with anti–IFN-γ and –TNF-α antibodies. Samples were run on an Aurora spectral flow cytometer (Cytek) before data analysis via OMIQ (https://www.omiq.ai/).

Effect of semaglutide on survival in an autochthonous KRAS-driven murine lung cancer model. *Kras^LSL-G12D^*; *Trp53^fl/fl^* (KP) mice (*55*) (The Jackson Laboratory, 032435) were fed a high-fat Western diet (60% kcal fat; Research Diets, D12492) with sugar water (23.1 g/L D-fructose + 18.9 g/L D-glucose) ad libitum for 13 weeks to induce DIO. Lung tumors were initiated via intratracheal adenoviral Cre recombinase (Ad-Cre; 2.5 × 10^7^ PFU; University of Iowa Viral Core) (56–59).Tumor-bearing DIO-KP mice were randomized to receive intraperitoneal semaglutide (60 nmol/kg/0.25 μg/gm body weight, 3 times per week; S9697, Selleck Chemicals) or saline (pH 7.4; Cytiva) (60). Imaging was conducted through the Oncology Precision Therapeutics and Imaging Core (OPTIC) at the Columbia University Herbert Irving Comprehensive Cancer Center. High-resolution μCT was performed to assess lung tumor burden, as previously described (61).

### Effects of GLP-1RA on survival outcomes in 2 retrospective clinical cohorts.

Two distinct single-center clinical cohorts were manually curated and analyzed.

In a postsurgical cohort, medical charts for patients undergoing surgical resection at the Roswell Park Cancer Center (RPCC) between 2015 and 2024 were manually curated. Patients were included for analysis if they had (a) histologically confirmed NSCLC and (b) a BMI greater than 25. Patients were defined as GLP-1RA users if they had GLP-1RA prescribed for more than 6 months during the period between resection and any event (defined as disease recurrence, death, or loss to follow-up).

In an advanced-disease cohort, medical charts for patients undergoing treatment for advanced or metastatic lung cancer at the RPCC between 2015 and 2023 were manually curated. Patients were included for analysis if they had (a) histologically confirmed advanced or metastatic NSCLC, (b) received at least 3 doses of an anti–PD-(L)1 and/or anti–CTLA-4 ICI (c), received no definitive local therapy (surgery or radiotherapy), and (d) had a BMI greater than 25. Patients were again defined as GLP-1RA users if they had GLP-1RA prescribed for more than 6 months during the period between the start of ICI treatment and any event (defined as disease progression, death, or loss to follow-up).

In both cohorts, patient demographics, including age, sex (male or female), race (White and non-White), smoking status (current, former, never smokers), and BMI were collected.

Visceral fat indexes and abdominal circumference were not routinely available in this retrospective database. For the surgical cohort, RFS was calculated from the date of resection until disease recurrence, death, or censoring at loss to follow-up. For the advanced-disease cohort, PFS was used, calculated from the date of treatment initiation until disease progression, death, or censoring at loss to follow-up. OS was defined from the point of resection in the postsurgical cohort and start of treatment in the advanced-disease cohort until the date of death or censoring at loss to follow-up.

### Statistics.

In the retrospective cohort analysis, results are reported as RFS, PFS, and OS as appropriate to the clinical scenario. To reduce confounding from baseline differences in small sample sizes, propensity scores were initially estimated using logistic regression with GLP-1RA use as the dependent variable. Propensity score matching was subsequently performed using greedy nearest-neighbor matching with a fixed control-to-treated ratio of 10:1 and a caliper width of 0.2 on the propensity score. The matching procedure was implemented in SAS version 9.4 (IBM) using PROC PSMATCH. Survival analysis was done using Cox’s proportional hazards regression on the matched cohort, accounting for the matched sets through stratification or clustering. Group differences were assessed using independent *t* tests and χ^2^ tests. All statistical tests were 2-sided with a level of significance of 5%. OS and PFS analyses were conducted using Cox’s proportional hazards modeling for univariate models. The statistics for survival analysis are expressed in terms of HR, and 95% CI with a 1-sided *P* value at a 5% level of significance. For animal model studies and in vitro experiments, the group differences were calculated using *t* tests or 2-way ANOVA tests. Statistical analyses were performed using SPSS version 28.0.1.0 (IBM) or GraphPad Prism version 9. Flow cytometry analysis was performed using OMIQ.

### Study approval.

The retrospective study protocol was reviewed and approved by the Institutional Review Board of the RPCC (BDR 115119), and individual patient consents were waived. All animal experiments conducted were reviewed and approved by the Institutional Animal Care and Use Committee (IACUC) of RPCC (protocols 1487 and 1349) or by Columbia University IACUC guidelines (protocol AABV8661).

### Data availability.

RPCC IRB protocols prohibit the sharing of clinical data to protect patient privacy. The data from mouse experiments are available in the Supporting Data Values file. Flow cytometry data will be made available as raw data files upon request. Gene sequencing data have been deposited in the NCBI Gene Expression Omnibus (GEO GSE302656).

## Author contributions

SY, BF, JB, and VRS contributed to the conceptualization of the study. SY and BF supervised the clinical aspects, while JB and VRS guided the translational components. AGP, SK, and YV curated the clinical data. AGP performed formal analysis of the clinical cohorts. AGP, MB, KR, and RVR carried out animal experiments. RJS and DW conducted and analyzed flow cytometry experiments. HHH and SR performed and supervised RNA sequencing analyses. JO and VRS contributed data and results from the autochthonous model. AGP, BF, and JB wrote the original draft of the manuscript, with data visualization by AGP, RJS, and SR. All authors reviewed and approved the final manuscript. Both AGP and BF are designated as co–first authors, as each contributed to equally critical aspects of the manuscript — AGP led most of the analysis, conducted the mouse experiments, and prepared the initial draft, while BF was primarily responsible for conceptualization and revision of the manuscript. AGP is listed first due to academic junior status and alphabetical order.

### Submission declaration.

A part of this study was submitted and presented at the Academic Surgical Congress – Annual Meeting 2025. This manuscript or any part of it is not under consideration for publication elsewhere.

## Supplementary Material

Supplemental data

ICMJE disclosure forms

Supporting data values

## Figures and Tables

**Table 1:**
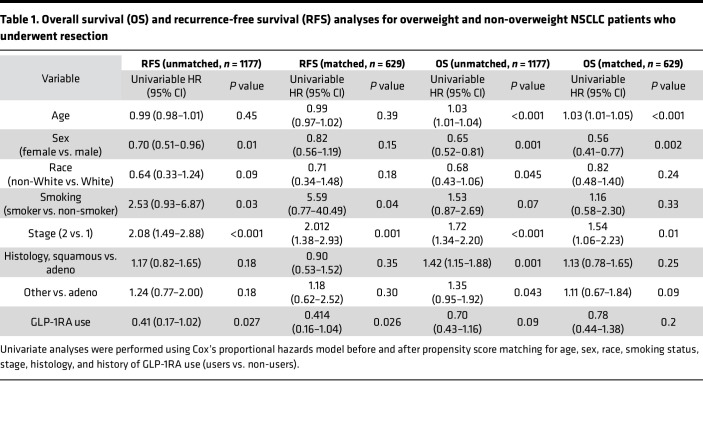
Overall survival (OS) and recurrence-free survival (RFS) analyses for overweight and non-overweight NSCLC patients who underwent resection

**Table 2:**
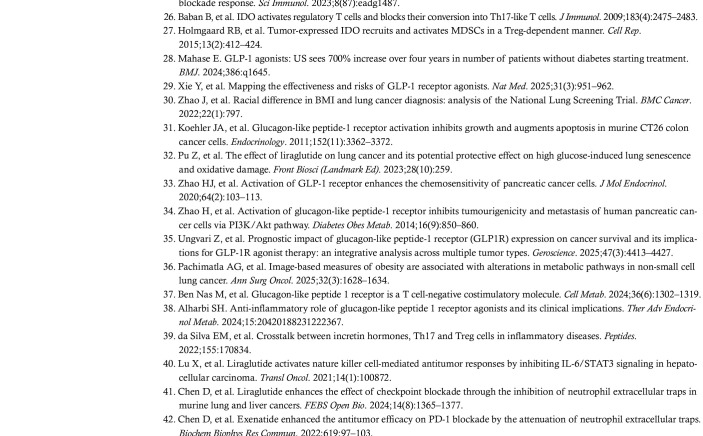
Overall survival (OS) and progression-free survival (PFS) analyses for overweight and non-overweight NSCLC patients on immune checkpoint inhibitors

## Version 1 08/26/2025

In-Press Preview

## Version 2 10/08/2025

Electronic publication
