# Supplementary material for: Glucagon-like peptide-1 receptor agonism improves lung cancer outcomes and tumor growth control
Source: JCI Insight. 2025 Aug 26;10(19):e195484. doi: 10.1172/jci.insight.195484 (PMC12513478; doi:10.1172/jci.insight.195484)
Supplement: Supplemental data [file jciinsight-10-195484-s186.pdf]

Supplemental Table 1

Characteristic	Unmatched cohort (n = 1177)	No GLP-1 RA (n = 1106)	GLP-1 RA (n = 71)	p-value	Matched cohort (n = 629)	No GLP-1 RA (n = 560)	GLP-1 RA (n = 69)	p-value
**Mean age (± SD)**	68.76 ± 8.37	68.90 ± 8.43	66.56 ± 7.03	**0.02**	67±8.06	67.02±8.19	66.83 ± 6.95	0.84
**Sex**				0.46				0.69
**Male**	517 (43.9%)	489 (44.2%)	28 (39.4%)		262 (41.65%)	235 (41.96%)	27 (39.13%)	
**Female**	660 (56.1%)	617 (55.8%)	43 (60.6%)		367 (58.35%)	325 (58.04%)	42 (60.87%)	
**Race**				0.29				0.69
**White**	1068 (90.7%)	1006 (91%)	62 (87.3%)		556 (88.39%)	496 (88.57%)	60 (86.96%)	
**Non-White**	109 (9.3%)	100 (9%)	9 (12.7%)		73 (11.61%)	64 (11.43%)	9 (13.04%)	
**Smoking status**				1				1.00
**Non-smoker**	82 (7%)	77 (7%)	5 (7%)		40 (6.36%)	36 (6.43%)	4 (5.8%)	
**smoker**	1095 (93%)	1029 (93%)	66 (93%)		589 (93.64%)	524 (93.57%)	65 (94.20%)	
**Mean BMI (± SD)**	30.93 ± 5.03	30.66 ± 4.81	35.07± 6.42	**0.001**	33.33±5.06	33.18±4.98	34.53±5.55	0.059
**Clinical stage**				0.41				0.88
**I**	970 (82.4%)	908 (82.1%)	62 (87.3%)		477 (75.83%)	425 (75.89%)	52 (75.36%)	
**II**	193 (16.4%)	184 (16.6%)	9 (12.7%)		152 (24.17%)	135 (24.11%)	17 (24.64%)	
**III**	14 (1.2%)	14 (1.3%)	0					
**Histology**				0.97				0.99
**Adenocarcinoma**	669 (56.8%)	628 (56.8%)	41 (57.7%)		376 (59.78%)	336 (60%)	40 (57.97%)	
**Squamous cell carcinoma**	345 (29.3%)	325 (29.4%)	20 (28.2%)		166 (26.39%)	147 (26.25%)	19 (27.54%)	
**Other**	163 (13.8%)	153 (13.8%)	10 (14.1%)		87 (13.83%)	77 (13.75%)	10 (14.49%)	

Supplemental Table 1. Patient characteristics of the Clinical Cohort A. Patient demographics (age, sex, race, smoking status, BMI) and disease/treatment characteristics (stage, histology) are given as mean/count and standard deviation (SD)/percentage. Overall distributions and group distributions, when split into GLP-1 RA users vs. nonusers, are provided. Group distributions of the full unmatched cohort and the post-propensity score matched cohort were compared using an independent t-test for continuous variables and chi-square tests for all other variables. P-value < 0.05 was considered statistically significant.

BMI: body mass index.

Supplemental Table 2

Characteristic	Unmatched cohort (n = 300)	No GLP-1 RA (n = 290)	GLP-1 RA (n = 10)	p-value	Matched cohort (n = 89)	No GLP-1 RA (n = 79)	GLP-1 RA (n = 10)	p-value
**Mean age (± SD)**	65.76 ± 9.28	65.97 ± 9.30	59.5 ± 6.32	**0.03**	60.39 ± 8.44	60.51 ± 8.70	59.50 ± 6.33	0.71
**Sex**				1				1
**Male**	149 (49.7%)	144 (49.7%)	5 (50%)		45 (50.56%)	40 (50.63%)	5 (5%)	
**Female**	151 (50.3%)	146 (50.3%)	5 (50%)		44 (49.44%)	39 (49.37%)	5 (5%)	
**Race***				0.49				1
**White**	271 (90.3%)	263 (90.7%)	8 (80%)		72 (80.90%)	64 (81.01%)	8 (80%)	
**Non-White**	28 (9.3%)	26 (9%)	2 (20%)		17 (19.10%)	15 (18.99%)	2 (20%)	
**Smoking status**				0.67				0.87
**current smoker**	123 (41%)	118 (41.7%)	5 (50%)		42 (47.19%)	37 (46.84%)	5 (50%)	
**former smoker**	161 (53.7%)	156 (53.8%)	5 (50%)		45 (50.56%)	40 (50.63%)	5 (50%)	
**never smoker**	16 (5.3%)	16 (5.5%)	0		2 (2.25%)	2 (2.53%)	00	
**Diabetes Mellitus**								
**Yes**	83 (27.5%)	73 (25.17)	10 (100%)		25 (28.09%)	15 (18.99%)	10 (100%)	
**No**	217 (72.4%)	217 (84.83%)	0 (0%)		64 (71.91%)	64 (71.91%)	0 (0%)	
**Mean BMI (± SD)**	30.37 ± 4.9	30.35 ± 4.9	31.2 ± 4.05	0.53	30.50 ± 4.49	30.41± 4.55	31.20± 4.05	0.409
**Histology**				0.27				0.93
**Adenocarcinoma**	180 (60%)	176 (60.7%)	4 (40%)		40 (44.94%)	36 (45.57%)	4 (40%)	
**Squamous cell carcinoma**	83 (27.7%)	78 (26.9%)	5 (50%)		40 (44.94%)	35 (44.30%)	4 (50%)	
**Other**	37 (12.3%)	36 (12.4%)	1 (10%)		9 (10.11%)	8 (10.13%)	1 (10%)	
**Stage**				0.45				1.00
**III**	64 (21.3%)	61 (21%)	3 (30%)		25 (28.09%)	22 (27.85%)	3 (30%)	
**IV**	236 (78.7%)	229 (79%)	7 (70%)		64 (71.91%)	57 (72.15%)	7 (70%)	
**Mode of therapy**				0.75				0.75
**Immunotherapy**	158 (52.7%)	152 (52.4%)	6 (60%)		42 (47.19%)	38 (48.10%)	4 (40%)	
**Chemo-immunotherapy**	142 (47.3%)	138 (47.6%)	4 (40%)		47 (52.81%)	41 (51.90%)	6 (60%)	
**PDL1 expression**				0.5				0.15
**<1**	69 (26.6%)	67 (26.9%)	2 (20%)		22 (27.8%)	20 (29%)	2 (20%)	
**1-50**	81 (31.3%)	79 (31.7%)	2 (20%)		31 (39.2%)	29 (42%)	2 (20%)	
**>50**	109 (42.1%)	103 (41.4%)	6 (60%)		26 (32.9%)	20 (29%)	6 (60%)	

Supplemental Table 2: Patient characteristics of the clinical cohort B. Patient demographics (age, sex, race, smoking status, BMI) and disease/treatment characteristics (histology, first-line regimen, PD-1 expression, T2DM status) are given as mean/count and standard deviation (SD)/percentage. Overall distributions as well as group distributions when split into GLP-1 RA vs. not on GLP-1 RA are provided. Group distributions of the full unmatched cohort and the post-propensity score matched cohort were compared using an independent t-test for age and chi-square tests for all other variables. P-value < 0.05 was considered statistically significant

*ICI = Immune checkpoint inhibitor, BMI = Body mass index, T2DM = Type 2 Diabetes mellitus

Supplemental Figure 1

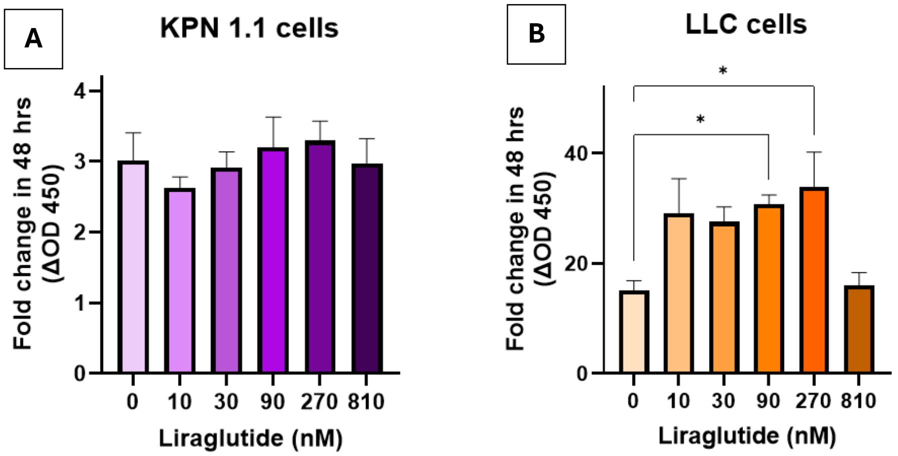

Supplemental Figure 1. Effects of liraglutide on in vitro tumor cell proliferation. The indicated cell lines were grown in vitro and subjected to the CCK-8 proliferation assay in the presence of various liraglutide concentrations. Shown are mean proliferation values +/-SD for the KPN1.1 **(A)** and LLC cell lines **(B)** from one of two independent experiments**.** *P<0.05 by t test.

Supplemental Figure 2

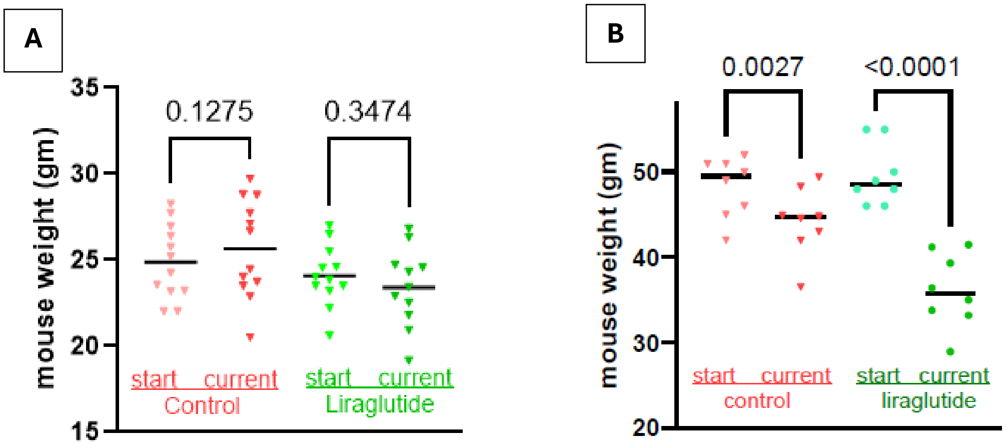

Supplemental Figure 2. Weight change in tumor-bearing mice treated with liraglutide or a PBS control. Obese C57BL/6 mice were challenged with s.c. KPN1.1 tumors and treated with liraglutide or PBS as described in Figure 2. Control mice had slight weight gain in normal-weight mice while liraglutide reduced the weight. Liraglutide-treated obese mice with higher weight loss than the control mice, as mice in both groups experienced significant weight loss **(A, B).** Numbers represent the P values for the indicated comparisons (t test).

Supplementary Figure 3

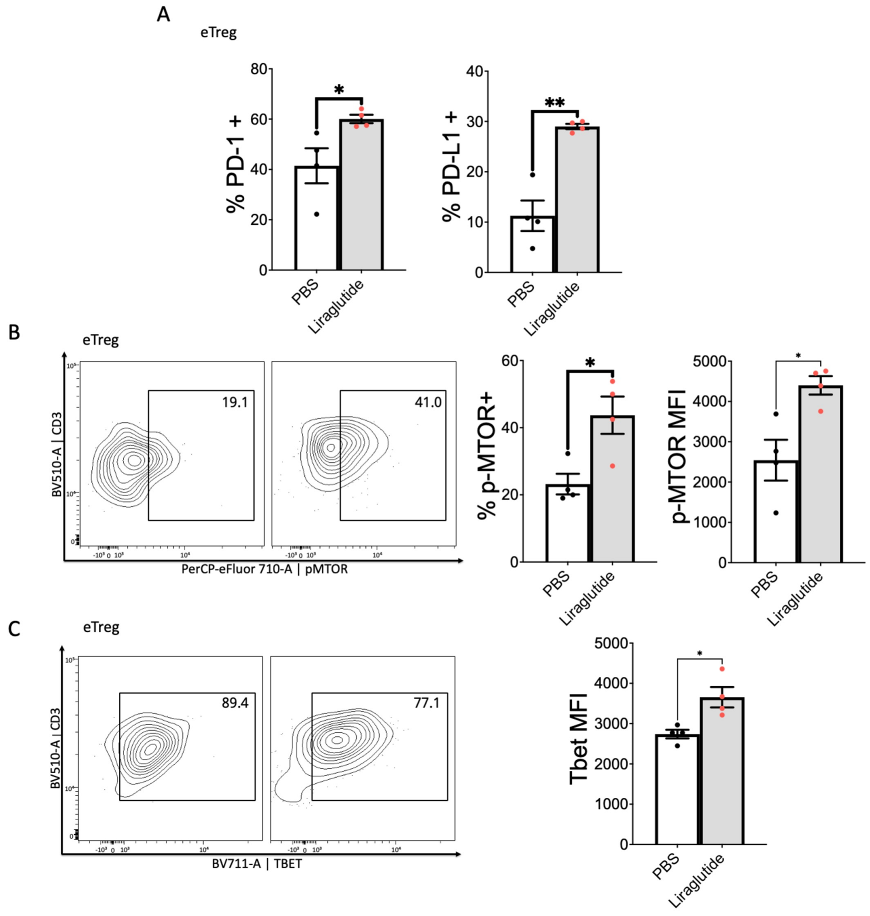

Supplemental Figure 3. Characterizing the impact of GLP-1Ra treatment on tumor-infiltrating Treg populations and phenotypes. Tumor tissues were recovered from the mice described in Figure 3, and, after enzymatic and mechanical digestion, single-cell suspensions were generated. Immunostaining followed by spectral flow cytometry analysis revealed the effects of liraglutide on Treg cells. Shown are the mean +/-SEM results from one of two independent experiments and representative flow plots (n=5 per group/trial). *$p < 0.05$, ***<0.01 by t test.

Supplementary Figure 4

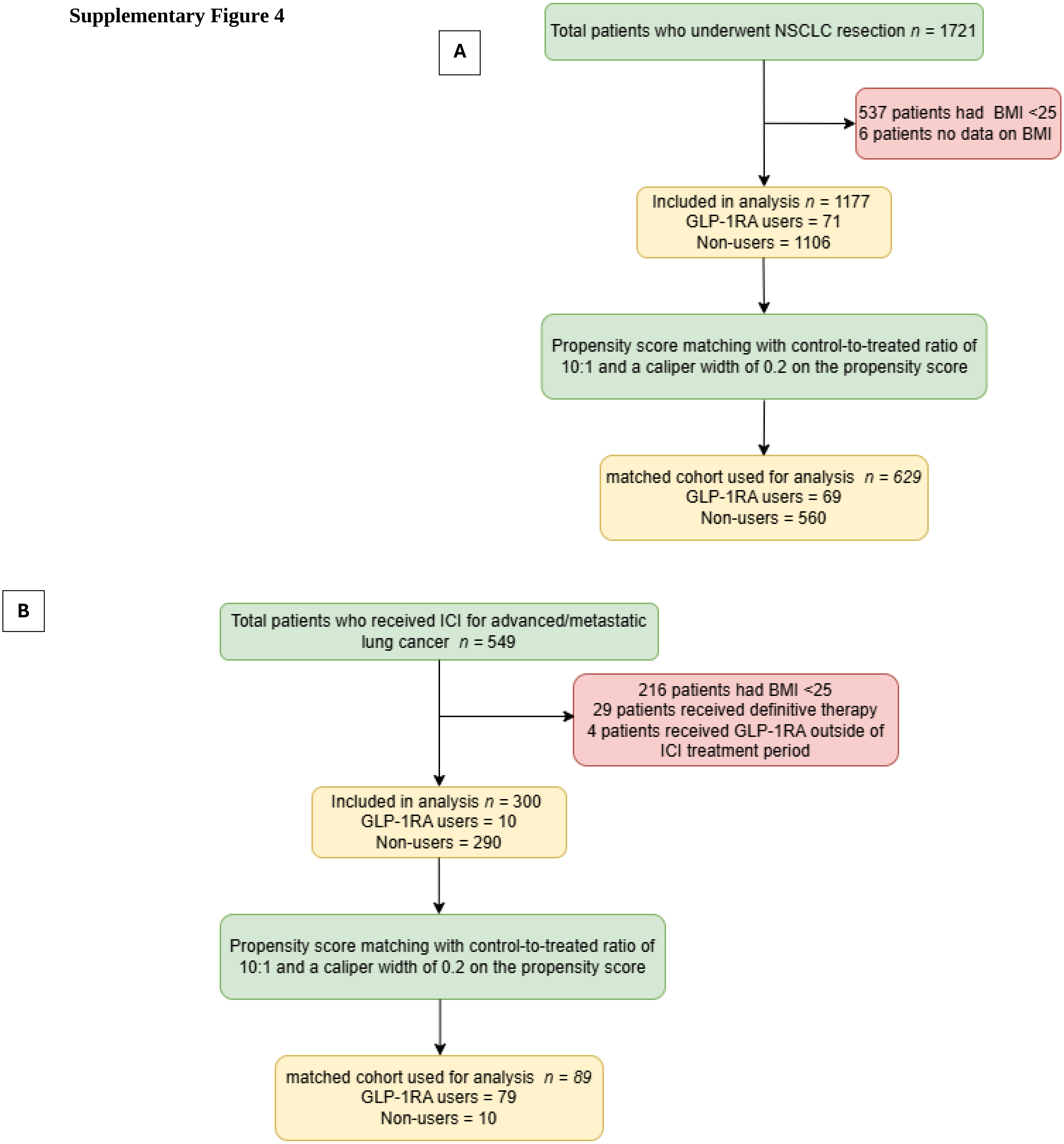

Supplemental Figure 4. Flowchart showing the Patient Cohort Selection. This flowchart illustrates the process of patient selection and analysis within both the retrospective clinical cohorts. It details the number of patients assessed at each stage, including initial screening, application of inclusion/exclusion criteria, and final inclusion in the study.
